# Generating Defective Epoxy Drop Images for Die Attachment in Integrated Circuit Manufacturing via Enhanced Loss Function CycleGAN

**DOI:** 10.3390/s23104864

**Published:** 2023-05-18

**Authors:** Lamia Alam, Nasser Kehtarnavaz

**Affiliations:** Department of Electrical and Computer Engineering, University of Texas at Dallas, Richardson, TX 75080, USA

**Keywords:** vision-based die attachment inspection, synthesized defective epoxy drop images, enhanced loss function CycleGAN

## Abstract

In integrated circuit manufacturing, defects in epoxy drops for die attachments are required to be identified during production. Modern identification techniques based on vision-based deep neural networks require the availability of a very large number of defect and non-defect epoxy drop images. In practice, however, very few defective epoxy drop images are available. This paper presents a generative adversarial network solution to generate synthesized defective epoxy drop images as a data augmentation approach so that vision-based deep neural networks can be trained or tested using such images. More specifically, the so-called CycleGAN variation of the generative adversarial network is used by enhancing its cycle consistency loss function with two other loss functions consisting of learned perceptual image patch similarity (LPIPS) and a structural similarity index metric (SSIM). The results obtained indicate that when using the enhanced loss function, the quality of synthesized defective epoxy drop images are improved by 59%, 12%, and 131% for the metrics of the peak signal-to-noise ratio (PSNR), universal image quality index (UQI), and visual information fidelity (VIF), respectively, compared to the CycleGAN standard loss function. A typical image classifier is used to show the improvement in the identification outcome when using the synthesized images generated by the developed data augmentation approach.

## 1. Introduction

In integrated circuit (IC) manufacturing, vision- or camera-based inspection systems are often used to enable automatic inspection of defects encountered during IC production. There are three primary components associated with such systems: a camera sensor, a computer running the inspection algorithms, and a sorter to separate defective and non-defective ICs. In this paper, defective epoxy drop images for die attachment are studied using die substrate images captured by a camera.

**Figure 1:**
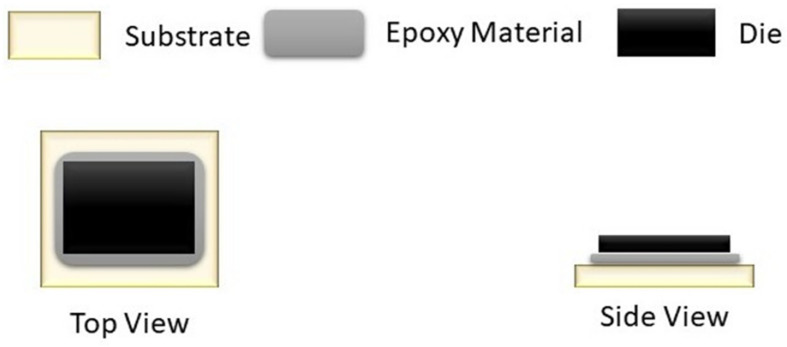
Epoxy die attachment.

Die attachment is a crucial step in the production of ICs. It is also referred to as die bonding or die mounting and involves attaching a silicon wafer die to a die pad or a substrate. Adhesive die attachments are most widely used due to their low cost [1]. In order to create a bond between a die and a substrate, an adhesive die attachment, as its name implies, uses an adhesive or epoxy. An epoxy die attachment equipment/die bonder is normally used to attach a die to its substrate using epoxy adhesive [2]. As illustrated in Figure 1, the die is set on top of the epoxy drop that is placed on the substrate and is bonded by heating.

A difficult aspect of die attachment is attaching the die to the substrate with the least amount of epoxy possible. An excessive amount of epoxy could cause the die or chip to tilt or overflow the substrate, compromising the stability of the entire IC package. On the other hand, an inadequate amount of epoxy could cause a bond line to become too thin, creating a weak bond with insufficient mechanical strength, which could lead to die cracking or die lifting. It is therefore necessary to inspect epoxy drops for proper die attachment. Due to the time and cost involved in conducting the inspection manually, the inspection process has been automated in modern IC manufacturing. The inspection process needs to be computationally efficient with high accuracy so that it is deployable in an actual production line.

A vision-based deep learning system can be utilized to conduct the inspection process automatically by examining images of an epoxy drop to assess whether a proper amount has been used. Depending on predefined quality standards, the system would accept or reject the die in real time. The use of modern deep neural networks requires the availability of a very large number of images with both adequate and inadequate amounts of epoxy. In practice, only a few images with inadequate amounts of epoxy, named defective or rejected epoxy images, are available since such cases occur quite infrequently. A good performance is normally reached when the training data are equally balanced for non-defective and defective image samples. Furthermore, although the lack of defective epoxy images can be mitigated by using conventional data augmentation techniques (such as cropping, rotating, flipping, and translating) [3], these techniques do not provide the image diversity needed for adequate training or testing of deep neural networks. The testing situation arises when only non-defective images are used for training a deep neural network and the generated defective images are used to test the trained network.

Generative adversarial network (GAN) models are being increasingly used for data augmentation purposes [4]. Many variations of GANs have been introduced in recent years. A variation of a GAN called CycleGAN [5] has been used to synthesize realistic images in different applications. In this paper, the CycleGAN standard loss function is modified or enhanced by utilizing learned perceptual image patch similarity (LPIPS) [6] and a structural similarity index metric (SSIM) [7] in order to generate more realistic defective or rejected epoxy images. Different combinations of LPIPS and the SSIM with/without the standard loss function are examined.

A data augmentation method for generating high-quality rejected epoxy images is introduced in this paper by enhancing the standard loss function CycleGAN via incorporating LPIPS and SSIM image quality metrics. This augmentation approach enables vision-based deep neural networks to be trained or tested more effectively by having equal numbers of defective and non-defective images. It is further shown that this data augmentation of synthesized defective images leads to improved identification outcomes.

The rest of the paper is organized as follows. Section 2 provides a review of previous works related to GAN models for data augmentation applications. An overview of CycleGAN is then covered in Section 3 together with the introduced enhanced loss function to address the die attachment problem of interest here. The experimental results in terms of quantitative evaluation metrics are then presented in Section 4 for the CycleGAN standard loss function and the enhanced loss function. The paper is finally concluded in Section 5.

## 2. Previous Works on Data Augmentation Using a GAN

This section provides a summary of data augmentation performed for different applications using a GAN. A GAN was used to produce many versions of an image in [8]. A review of medical image augmentation papers using a GAN was covered in [9]. A pixel-level image augmentation technique was developed in [10] based on image-to-image translation with a GAN. It was trained on a surface defect dataset of magnetic particle images to generate synthesized image samples. A variation of CycleGAN named AttenCGAN was proposed in [11] to synthesize electrical commutators and surface images with artificial defects to increase the number of image samples. A defect transfer GAN (DT-GAN) was developed in [12] to produce realistic surface defect images. The Mask2Defect GAN was suggested in [13] to create surface defect images obtained from an automobile part stamping plan. A region- and strength-controllable GAN for creating synthesized defects in metal surfaces was also proposed in [14] based on the idea of image inpainting. To produce high-quality defect images, a so-called relative mean generative adversarial network (TARGAN) was introduced in [15] using a metal gear surface defect image dataset and a hot-rolling strip defect image dataset. In [16], an MAS-GAN-based model was proposed for the production of industrial defect images by combining an attention mechanism and a data augmentation module. A framework called DefectGAN was introduced in [17] by using a compositional-layer-based architecture to generate realistic defect images. For data augmentation of surface defects on hot-rolled steel strips, three GANs were trained in [18], a new GAN called a contrastive GAN was proposed in [19], and a semi-supervised learning (SSL) defect classification approach based on two different networks of a categorized generative adversarial network (GAN) and a residual network was proposed in [20]. In order to produce defect images using a large number of defect-free images of commutator cylinder surfaces from industrial sites, a generation technique known as the surface defect generation adversarial network (SDGAN) was introduced in [21]. GAN models were also utilized for unsupervised surface inspection in [22], for anomaly detection on structured and arbitrary textured surfaces in [23], for Mura defect classification in thin-film transistor liquid crystal display (TFT-LCDs) in [24], for enhancing the quantity and quality of images of fabric defects in [25], for the autonomous design of architectural shape sketches in [26], and for establishing the probabilistic correlations of quasi-static responses of bridges in [27].

More recently, GANs have been used for IC manufacturing applications. A multi-scale GAN with a transformer (MST-GAN) as a semi-supervised deep learning network was developed for IC metal package samples in [28]. An IC solder joint inspection approach was suggested in [29] based on a GAN model and statistical training. In [30], a GAN model was used to generate pseudo-defective wafer die images from real defective images. A GAN-based image generation technique for organic light-emitting diode (OLED) panel defect images was discussed in [31].

In this paper, our objective is to produce realistic defective or rejected epoxy substrate images based on the few available such images. To meet this objective, we make use of a large number of defect-free or good epoxy substrate images that are available in order to generate a large number of defective or rejected epoxy substrate images by using CycleGAN to translate non-defective or good images to rejected images. For this purpose, we enhanced the CycleGAN cycle consistency loss function by incorporating other loss functions, which are discussed in Section 3.

## 3. Generating Synthesized Defective Images via CycleGAN

The most widely used conditional generative adversarial network for the purpose of unpaired image-to-image translation is called CycleGAN [5]. A typical CycleGAN learns the mapping between two distributions via optimization of an objective function by using two generators and two discriminators. Two losses are incorporated into the CycleGAN optimization framework: adversarial loss and cycle consistency loss. The adversarial loss measures the difference between the generated images and the target images according to the original GAN design [4], and the cycle consistency loss is used to avoid conflicts between the learnt mappings. In our problem, we generate synthesized defective or rejected epoxy drop substrate images from non-defective or good epoxy drop substrate images. Despite the fact that the CycleGAN generates realistic synthetic images, ambiguity mapping occurs when a domain with rich information (i.e., good epoxy drop substrate images) is translated into a domain with relatively weak information (i.e., rejected epoxy drop substrate images). This ambiguity mapping is addressed in this paper by adding the loss functions of LPIPS and the SSIM to the standard cycle consistency loss function of the generator network. More details of these loss functions are stated later in this section.

**Figure 2:**
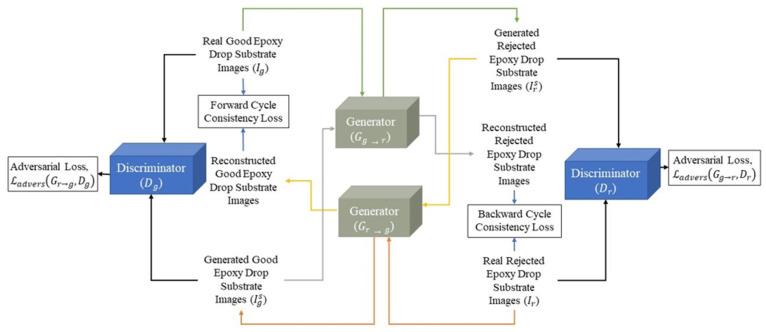
CycleGAN framework used for our data augmentation.

Two generator networks and two discriminator networks make up the CycleGAN architecture [5]. Adversarial training is carried out on the networks against one another. The generators’ objective is to convert an image from one domain to another. The discriminators’ objective is to distinguish between real and synthesized images in their respective domains. Figure 2 demonstrates the CycleGAN framework that we utilized to achieve data augmentation of defective or rejected epoxy drop substrate images. The data augmentation model contains the two mapping functions $G_{g\to r}:Good\to Rejected$ and $G_{r\to g}:Rejected\to Good$, and the associated adversarial discriminators $D_{r}$ and $D_{g}$. Here, $D_{r}$ distinguishes between real rejected epoxy drop substrate images {$I_{r}$} and synthesized rejected epoxy drop substrate images {$I_{r}^{s}$} generated from real good epoxy drop substrate images {$I_{g}$}, and likewise $D_{g}$ distinguishes between {$I_{g}$} and {$I_{g}^{s}$}. The total loss function of the CycleGAN can be expressed as a summation of the adversarial losses ($L_{advers})$ and the cycle consistency loss ($L_{cyc})$:

(1) $$L\left(G_{g\to r},G_{r\to g},D_{g},D_{r}\right)=L_{advers}\left(G_{g\to r},D_{r}\right)+L_{advers}\left(G_{r\to g},D_{g}\right)+L_{cyc}\left(G_{g\to r},G_{r\to g}\right)$$

Adversarial losses make sure that the generated images appear realistic, and the cycle consistency loss reflects the difference between the original image and the reconstructed or transformed image. The aim is to solve the following optimization problem

(2) $$G_{\left(g\to r\right)}^{*},G_{\left(r\to g\right)}^{*}=arg\underset{G_{g\to r},G_{r\to g}}{\min}\underset{D_{g},D_{r}}{\max}L\left(G_{g\to r},G_{r\to g},D_{g},D_{r}\right)$$

More details of the loss functions are stated next.

**Adversarial loss function**: For both the mapping functions, the adversarial losses in [4] adopted by the standard Cycle GAN are used. For the mapping function $G_{g\to r}$ and its discriminator $D_{r}$, the optimization problem can be written as

(3) $$L_{advers}\left(G_{g\to r},D_{r},I_{g},I_{r}\right)=E_{i_{r}\sim p_{data}(i_{r})}[\log D_{r}(i_{r})]+E_{i_{g}\sim p_{data}(i_{g})}[\log\left(1-{D_{r}(G}_{g\to r}\left(i_{g}\right))\right)]$$

where $i_{r}\sim p_{data}\left(i_{r}\right)$ and $i_{g}\sim p_{data}\left(i_{g}\right)$ denote the distributions of $I_{r}$ and $I_{g}$, respectively, and 𝔼 denotes the expected value over all real data instances. Here, $G_{g\to r}$ attempts to generate images $G_{g\to r}\left(i_{g}\right)$ that look like $I_{r}$ images, while $D_{r}$ attempts to distinguish between synthesized images $I_{r}^{s}\approx G_{g\to r}\left(i_{g}\right)$ and real images $I_{r}$. Similarly, for the mapping function $G_{r\to g}$ and its discriminator $D_{g}$, the optimization problem can be written as

(4) $$L_{advers}\left(G_{r\to g},D_{g},I_{r},I_{g}\right)=E_{i_{g}\sim p_{data}(i_{g})}[\log D_{g}(i_{g})]+E_{i_{r}\sim p_{data}(i_{r})}[\log\left(1-{D_{g}(G}_{r\to g}\left(i_{r}\right))\right)]$$

**Cycle consistency loss**: Cycle consistency loss converts images back to their original domain, i.e., $i_{g}\to G_{g\to r}\left(i_{g}\right)\to {G_{r\to g}(G}_{g\to r}\left(i_{g}\right)\approx i_{g}$, known as the forward cycle consistency loss, and $i_{r}\to G_{r\to g}\left(i_{r}\right)\to {G_{g\to r}(G}_{r\to g}\left(i_{r}\right)\approx i_{r}$, known as the backward cycle consistency loss. Cycle consistency loss is defined as the combination of the following losses:

(5) $$L_{Cycle}\left(G_{r\to g},G_{g\to r}\right)=L^{F\_Cycle}\left(G_{g\to r}\right)+L^{B\_Cycle}\left(G_{r\to g}\right)$$

where $L^{F\_Cycle}$ and $L^{B\_Cycle}$ represent the forward and backward cycle consistency losses, respectively. The cycle consistency loss makes sure that the features of the input images are preserved in the generated images. For the cycle consistency loss, it matters which loss function is used for the cycle consistency loss. In this work, we consider several loss functions separately and in combination for the cycle consistency loss in order to improve the CycleGAN performance for the die attachment problem of interest here. When combining the loss functions, they are normalized so that their contributions to the combined loss function are made equal. A description of the loss functions considered is presented next.

$L_{1}$ **loss function**: Additionally, referred to as mean absolute error (MAE) loss, measures the absolute distance between the generated image and the target image. In our case, it is obtained by taking the absolute value of the real good image $I_{g}$ and the reconstructed good image $G_{r\to g}(G_{g\to r}\left(i_{g}\right))$. It can be expressed as follows:

(6) $$L_{L_{1}}^{F\_Cycle}\left(i_{g},{G_{r\to g}(G}_{g\to r}\left(i_{g}\right))\right)=E_{{i_{g}\sim p_{data}\left(i_{g}\right),}_{}}\left[{\left\|i_{g}-{G_{r\to g}(G}_{g\to r}\left(i_{g}\right))\right\|}_{1}\right]$$

Similarly, the absolute value of the real rejected image $I_{r}$ and the reconstructed good image $G_{g\to r}(G_{r\to g}\left(i_{r}\right))$ is obtained as follows:

(7) $$L_{L_{1}}^{B_{Cycle}}(i_{r},{G_{r\to g}(G}_{g\to r}\left(i_{g}\right)))=E_{i_{r}\sim p_{data}\left(i_{r}\right)}[{\left\|i_{r}-G_{g\to r}\left(G_{r\to g}\left(i_{r}\right)\right)\right\|}_{1}]$$

$L_{1}$ loss function reduces the absolute difference between the images. It is mostly used to capture low-frequency details or to enforce the accuracy of low frequencies. It has been used to compute the cycle consistency loss in the standard CycleGAN [5]. The quality of the generated images can be improved by combining this kind of loss function with another loss function, as discussed in [32].

$L_{2}$ **loss function:** Additionally, referred to as mean squared error (MSE), is obtained by squaring the difference between the generated image and the target image. In our case, it can be expressed as follows:

(8) $$L_{L_{2}}^{F_{Cycle}}(i_{g},{G_{r\to g}(G}_{g\to r}\left(i_{g}\right)))=E_{{i_{g}\sim p_{data}\left(i_{g}\right)}_{}}[{\left\|i_{g}-{G_{r\to g}(G}_{g\to r}\left(i_{g}\right))\right\|}_{2}^{2}]$$

(9) $$L_{L_{2}}^{B\_Cycle}(i_{r}{G_{r\to g}(G}_{g\to r}\left(i_{g}\right)))=E_{i_{r}\sim p_{data}(i_{r})}[{\left\|i_{r}-{G_{g\to r}(G}_{r\to g}\left(i_{r}\right))\right\|}_{2}^{2}]$$

In [33], $L_{1}$ and $L_{2}$ losses were compared and no discernible difference between them was found. However, according to [34], $L_{1}$ loss is preferred over $L_{2}$ loss because it promotes less blurring. Both of these losses represent pixel-wise losses. They consider pixel-by-pixel variations between the images. Even though the images being compared are comparable to the human visual system, there exists a loss in value. Since the computation is based on each pixel, it is not significantly affected if one shifts an image by just one pixel. The total loss value gradually rises as a result of the aggregation of each minor difference between the corresponding pixels of two images. Hence, adding some other loss function to the standard loss functions can help to improve the performance of the model.

**Structural similarity index metric (SSIM)**: This index has been extensively utilized to assess image quality [7] and has been used as loss function for numerous image processing applications [35,36] as well as for GAN-based solutions [32,37,38]. It was created under the presumption that the human visual system is extremely well suited for sifting through structural data in a visual input. The structural information degradation between a generated image and a corresponding input image is measured by the SSIM. Luminance, contrast, and structure are three sub-indices that make up the SSIM. Luminance is reflected in the local means, contrast in the local standard deviations, and structure in the local Pearson correlation between two images. For an input image x and a reconstructed image y, the SSIM is defined as follows:

(10) $$SSIM(x,y)=\frac{\left(2\mu_{x}\mu_{y}+C_{1})(2\sigma_{xy}+C_{2}\right)}{{(\mu_{x}^{2}+\mu_{y}^{2}+C}_{1})(\sigma_{x}^{2}+\sigma_{y}^{2}+C_{2})}$$

where $\mu_{x}$ and $\mu_{y}$ are the mean intensities, $\sigma_{x}$ and $\sigma_{y}$ are the variances, and $\sigma_{xy}$ is the covariance of images *x* and *y*. The constants $C_{1}$ and $C_{2}$ are used to prevent numerical singularity. More information on this index appears in [7]. Here, the SSIM is included as a loss function to produce visually acceptable images. This index can be expressed as follows:

(11) $$L_{SSIM}^{F\_Cycle}\left(i_{g},{G_{r\to g}(G}_{g\to r}\left(i_{g}\right))\right)=[1-SSIM\left(i_{g},{G_{r\to g}(G}_{g\to r}\left(i_{g}\right))\right)]$$

(12) $$L_{SSIM}^{B\_Cycle}\left(i_{r},{G_{g\to r}(G}_{r\to g}\left(i_{r}\right))\right)=[1-SSIM\left(i_{r},{G_{g\to r}(G}_{r\to g}\left(i_{r}\right))\right)]$$

**Figure 3:**
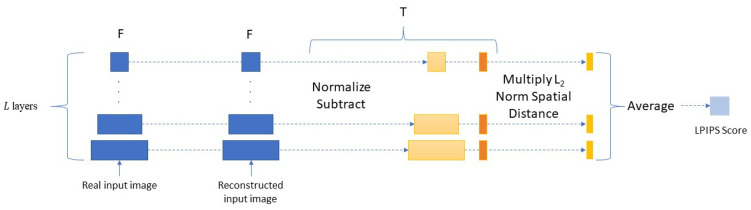
Network to compute LPIPS.

**Learned perceptual image patch similarity (LPIPS)**: LPIPS indicates how similar two images appear to the human eye. In essence, LPIPS determines how comparable the activations of two image patches are for a given network. Therefore, we use it here as a loss function. Figure 3 and Equations (13) and (14) show how a pertained network F is used to compute the LPIPS score between a real input image and a reconstructed image.

(13) $$L_{LPIPS}^{F\_Cycle}\left(i_{g},{G_{r\to g}(G}_{g\to r}\left(i_{g}\right))\right)=\sum_{l}{Τ}^{l}({\left\|F^{l}\left(i_{g}\right)-F^{l}\left({G_{r\to g}(G}_{g\to r}\left(i_{g}\right))\right)\right\|}_{2}^{2})$$

(14) $$L_{LPIPS}^{B\_Cycle}\left(i_{r},{G_{g\to r}(G}_{r\to g}\left(i_{r}\right))\right)=\sum_{l}{Τ}^{l}({\left\|F^{l}\left(i_{r}\right)-F^{l}\left({G_{g\to r}(G}_{r\to g}\left(i_{r}\right))\right)\right\|}_{2}^{2})$$

where F denotes the pertained network with l∈L layers for feature extraction, and Τ normalizes and scales the deep embedding to a scalar LPIPS score. Then, the $L_{2}$ distance is computed and averaged across the dimensions and layers of the network. For feature distances, the AlexNet [39] network is used here, which is more in line with the structure of the human visual cortex [6,40].

### Model Architecture and Training

**Figure 4:**
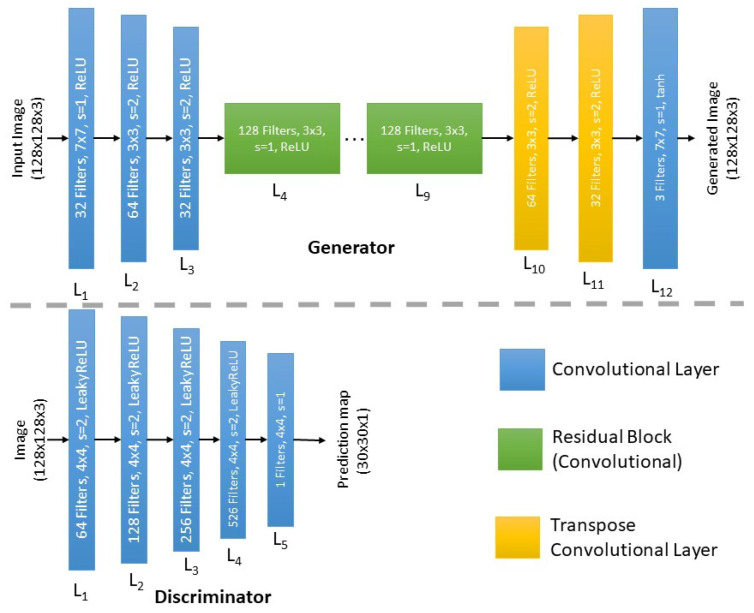
Architecture of the generator and discriminator of our implemented CycleGAN.

For the generator and discriminator, the same architecture described in the standard CycleGAN [5] is used here; see Figure 4. The generator feeds its 128 × 128 input image through three convolutional layers in succession, each of which causes the representation to become smaller with more channels. Afterwards, a set of six residual blocks follows, each with 128 filters. Transpose convolutional layers are used to further enhance the representation for the final image. Apart from the Tanh activation in the last layer for reconstruction, each layer is followed by instance normalization and a rectified linear unit (ReLU) as the activation function. The generated image is 128 × 128 in size. The Markovian discriminator (PatchGAN) [41] is utilized to determine if the image patches are real or synthesized for the discriminator. Five convolutional layers make up the discriminator, which is a fully convolutional network. In order to keep the size of the feature maps at 1/8, the stride is only set to 2 for the first four convolutional layers and the instance normalization along with Leaky ReLU are utilized as the activation function. To preserve the size of the feature maps, the stride of the final output layers is set to 1 and the filter number is set to 1 in order to produce a one-channel prediction map with values ranging from 0 to 1 for every pixel. The discriminator’s input is a real or synthesized image having a size of 128 × 128, and the output is 30 × 30 in size. In order to determine if a patch of the input image is real or synthesized, each output pixel corresponds to a patch of the input image.

After defining each component of the CycleGAN, the network is trained. A pseudo code of the training is shown in Algorithm 1. CycleGAN offers a clear advantage over utilizing unpaired data. However, our image-generating network is trained using the paired real good epoxy drop substrate images and real rejected epoxy drop substrate images in order to test the generated images in a uniform manner. The batch size is set to 1 with 200 epochs and *k* = 100, which is sufficient for convergence. The model is optimized using the Adam optimizer with $\beta_{1}=0.5$ and an initial learning rate of 0.0002 for the first 100 epochs, decreasing the learning rate linearly to 0 for the last 100 epochs. Our CycleGAN with different cycle consistency losses is implemented in Python (environment version 3.8.13) using the TensorFlow (version 2.9.1) and Pillow (version 9.2.0) libraries.

**Algorithm 1.** Cycle GAN Training1: for number of epochs do2: for k iterations do3:  Draw a minibatch of samples $\left\{i_{g}^{\left(1\right)},\ldots ,i_{g}^{\left(m\right)}\right\}$ from data distribution,
$p_{data}\left(i_{g}\right)$ domain $I_{g}$4:  Draw a minibatch of samples $\left\{i_{r}^{\left(1\right)},\ldots ,i_{r}^{\left(m\right)}\right\}$ from data distribution,
$p_{data}\left(i_{r}\right)$ domain $I_{r}$5:  Generate m synthetic samples

$I_{r}^{s}:I_{g}\to G_{g\to r}(i_{g})$



$I_{g}^{s}:I_{r}\to G_{r\to g}(i_{r})$

6:  Compute adversarial losses // Combination of discriminator loss on both real and fake images

$L_{advers}\left(G_{g\to r},D_{r},I_{g},I_{r}\right)=E_{i_{r}\sim p_{data}(i_{r})}[\log D_{r}(i_{r})]+E_{i_{g}\sim p_{data}(i_{g})}[\log\left(1-{D_{r}(G}_{g\to r}\left(i_{g}\right))\right)]$



$L_{advers}\left(G_{r\to g},D_{g},I_{r},I_{g}\right)=E_{i_{g}\sim p_{data}(i_{g})}[\log D_{g}(i_{g})]+E_{i_{r}\sim p_{data}(i_{r})}[\log\left(1-{D_{g}(G}_{r\to g}\left(i_{r}\right))\right)]$

7:  Update the discriminators $D_{g}$ and $D_{r}$

$\underset{D_{g}}{\max}L_{advers}\left(G_{r\to g},D_{g},I_{r},I_{g}\right)$



$\underset{D_{r}}{\max}L_{advers}\left(G_{g\to r},D_{g},I_{g},I_{r}\right)$

8:  Generate m cycle samples 

$I_{g}^{Cycle}:G_{g\to r}(i_{g})\to {G_{r\to g}(G}_{g\to r}(i_{g}))$



$I_{r}^{Cycle}:G_{r\to g}(i_{r})\to G_{g\to r}(G_{r\to g}(i_{r}))$

9:  Compute Cycle Consistency Loss

$L_{Cycle}\left(G_{r\to g},G_{g\to r}\right)=L^{F\_Cycle}\left(G_{g\to r}\right)+L^{B\_Cycle}\left(G_{r\to g}\right)$

/* Different loss functions separately and in combination were used to calculate the cycle consistency losses $L^{F\_Cycle}\left(G_{g\to r}\right)$ and $L^{B\_Cycle}\left(G_{r\to g}\right)$ */10:  Compute total generator loss

$L\left(G_{g\to r},G_{r\to g},D_{g},D_{r}\right)=L_{advers}\left(G_{g\to r},D_{r},I_{g},I_{r}\right)+L_{advers}\left(G_{r\to g},D_{g},I_{r},I_{g}\right)+\lambda L_{cyc}\left(G_{g\to r},G_{r\to g}\right)$

11:  Update the generators $G_{g\to r}$ and $G_{r\to g}$

$\underset{G_{g\to r},G_{r\to g}}{\min}L\left(G_{g\to r},G_{r\to g},D_{g},D_{r}\right)$



## 4. Experimental Results and Discussion

In this section, first we report our experiments to assess the quality of the generated images based on our introduced enhanced loss function CycleGAN. Then, we report our experiments using a typical image classifier (ResNet18) [42]) to show the identification outcomes with and without using the generated synthesized defective or rejected images.

Our experiments were performed on a server with the 64-bit Windows 10 operating system with two Intel^®^ Xeon^®^ 2.40 GHz CPUs and with two NVIDIA Tesla K40 m graphics cards having 256 GB RAM.

### 4.1. Quantative Evaluation of Generated Images

#### 4.1.1. Evaluation Metrics

Our goal is to create high-quality synthesized rejected epoxy drop substrate images. This requires quantitative quality evaluation metrics of the synthesized images. The metrics that are often used for this purpose include peak signal-to-noise ratio (PSNR) [7], the universal image quality index (UQI) [43], and visual information fidelity (VIF) [44]. The testing dataset consists of paired images of the same size.

**Peak signal-to-noise ratio (PSNR)**: Given its simplicity and ease of use, the PSNR is the most widely used metric for evaluating synthesized images. The PSNR indicates the difference in pixels between a synthesized and a real image. The quality of the resulting image improves with an increasing PSNR. Equation (15) indicates how the PSNR is computed:

(15) $$PSNR=20\log_{10}\left(\frac{{Peak\;Value}^{2}}{MSE}\right)$$

where Peak Value denotes the highest value in the image data, and for an 8-bit unsigned integer data type, it is 255. The mean squared error (MSE) between two images is given by

(16) $$MSE=\frac{1}{NN}\sum_{i=1}^{N}\sum_{j=1}^{N}{\left(x\left(i,j\right)-y\left(i,j\right)\right)}^{2}$$

with x and y representing, respectively, the real and the synthesized images of size N×N. Equation (15) reflects the absolute error in dB.

**Universal image quality index (UQI)**: The UQI compares generated synthesized and real images in terms of luminance, contrast, and structure, reflecting the characteristics of the human visual system. It corresponds to the special case of the SSIM when ${C_{1}=C}_{2}=0$ in Equation (10) and can be written as the product of three components of correlation, luminance distortion, and contrast distortion, as follows:

(17) $$UQI=\frac{\sigma_{xy}}{\sigma_{x}\sigma_{y}}\cdot \frac{2\bar{x}\bar{y}}{{\left(\bar{x}\right)}^{2}+{\left(\bar{y}\right)}^{2}}\cdot \frac{2\sigma_{x}\sigma_{y}}{\sigma_{x}^{2}+\sigma_{y}^{2}}$$

where $x=\{x_{i}|i=1,2,\ldots ,N\}$ and $y=\left\{y_{i}|i=1,2,\ldots ,N\right\}$ denote the real and the synthesized images, respectively, $\bar{x}=\frac{1}{N}\sum_{i=1}^{N}x_{i}$, $\bar{y}=\frac{1}{N}\sum_{i=1}^{N}y_{i}$, $\sigma_{x}^{2}=\frac{1}{N-1}\sum_{i=1}^{N}{{(x}_{i}-\bar{x})}^{2}$, $\sigma_{y}^{2}=\frac{1}{N-1}\sum_{i=1}^{N}{{(y}_{i}-\bar{y})}^{2}$, and $\sigma_{xy}=\frac{1}{N-1}\sum_{i=1}^{N}{(x}_{i}-\bar{x})(y-\bar{y}).$ The dynamic range of the UQI is [−1, 1]. The best value 1 is achieved if and only if for all $i=1,2,\ldots ,N,x_{i}=y_{i}$.

**Visual information fidelity (VIF)**: Visual information fidelity (VIF) is a full reference image quality assessment index based on natural scene statistics and the human visual system (HVS). The HVS is used to determine the accuracy of visual information, which includes factors such as the sharpness of edges, the accuracy of color representation, and the ability to detect subtle changes in contrast. VIF measures image fidelity by comparing the information recovered from a real image x with the information lost in a synthesized image y using the HVS. It is a straightforward ratio of the real and the generated images with a value between 0 and 1 and is defined as follows:

(18) $$VIF=\frac{HVS(y)}{HVS(x)}$$

#### 4.1.2. Dataset

**Figure 5:**
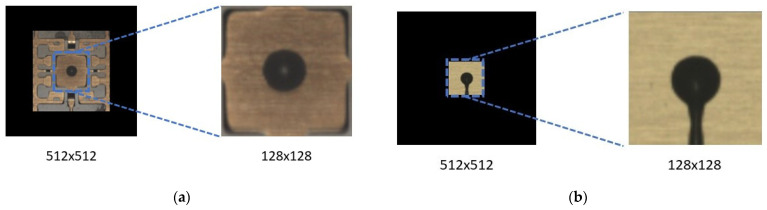
ROI for image generation.

**Figure 6:**
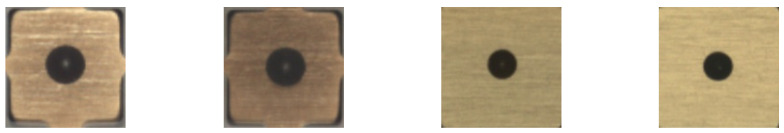
Samples images of non-defective or good epoxy drop substrate.

**Figure 7:**
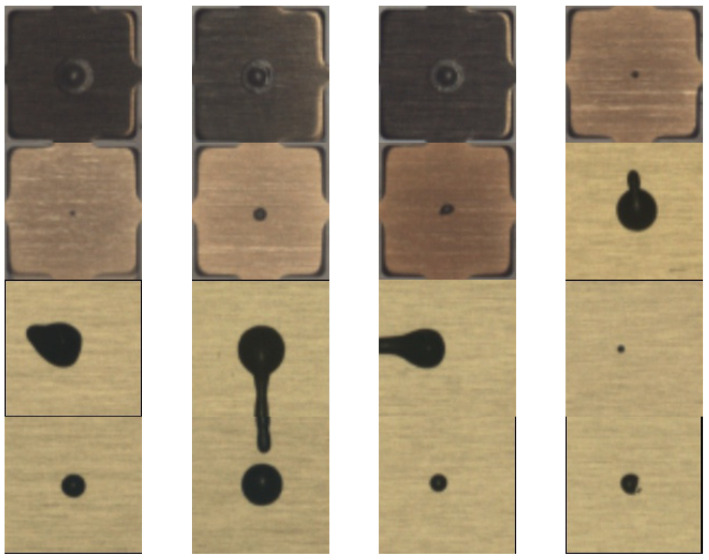
Defective or rejected images of epoxy drop substrate.

A dataset of O-shape epoxy drop substrate images was provided to us by Texas Instruments. In the dataset, there were 8850 good epoxy drop substrate images and only 16 rejected epoxy drop substrate images. As explained earlier, this is because defective patterns of epoxy drops rarely occur during production. To ready the dataset for processing, we cropped the region of interest (ROI), having a size of 128 × 128, from the images, as illustrated in Figure 5 (Figure 5a shows ROI cropping of non-defective or good epoxy drop substrate and Figure 5b shows ROI cropping of defective or rejected epoxy drop substrate). We selected 88 good epoxy drop substrate images with different lighting conditions/backgrounds and paired them with rejected epoxy drop substrate images. Some sample non-defective or good epoxy drop substrate images are shown in Figure 6. Additional defective or rejected epoxy drop substrate images were generated by rotation and vertical/horizontal flips for the experiments. Figure 7 shows the 16 rejected real epoxy drop substrate images.

#### 4.1.3. Results and Discussion

We started our experiments by training the model using the standard cycle consistency loss (i.e., $L_{1}$) along with the other loss functions (i.e., $L_{2}$, SSIM and LPIPS) separately as well as in combination. Then, we generated realistic synthesized images after training the model.

**Figure 8:**
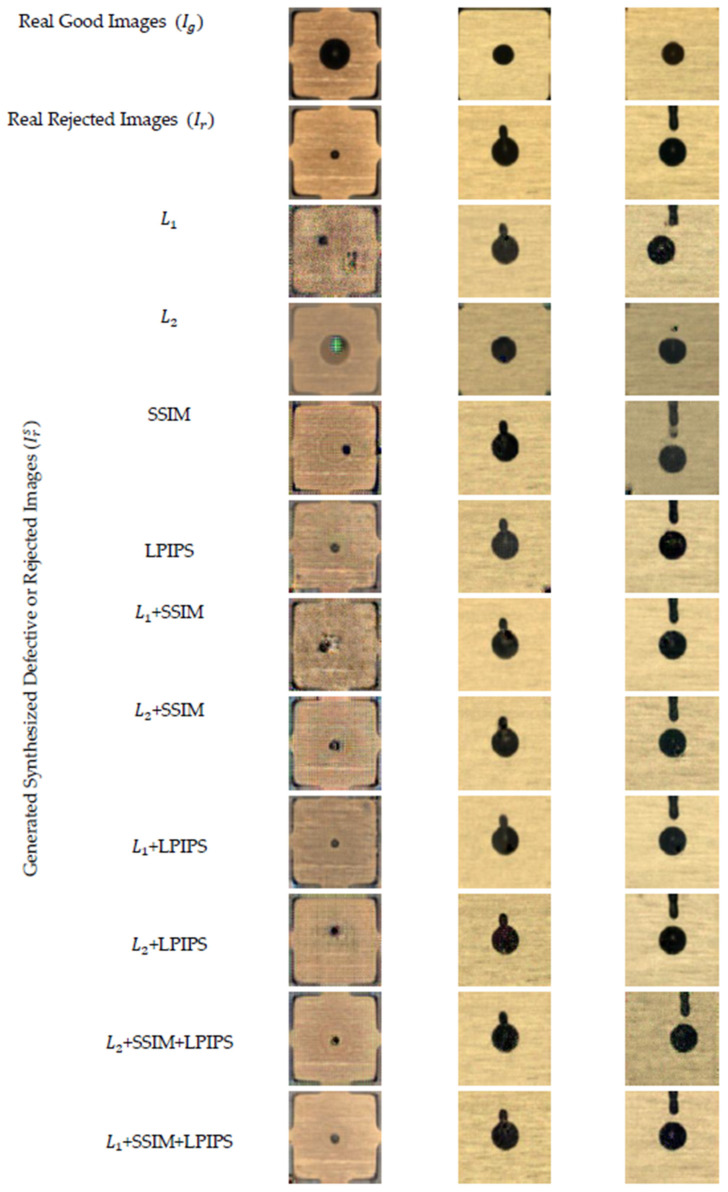
Sample images of generated rejected epoxy drop substrate using different loss functions as the cycle consistency loss function.

Figure 8 shows some sample outcomes of the generated synthesized rejected epoxy drop substrate images using different loss functions as the cycle consistency loss, separately and in combination with the CycleGAN standard loss function. Table 1 shows the evaluation metrics for different loss functions as the cycle consistency loss ($L_{Cycle})$. As the dataset utilized was made up of paired image data, all the generated synthesized rejected images (i.e., $I_{r}^{s})$ exhibited a relatively uniform reference material or real rejected epoxy drop substrate images (i.e., $I_{r}$). This table shows the averages and standard deviations of the metrics for the rejected images $I_{r}$ and their generated counterpart, i.e., the generated synthesized images $I_{r}^{s}$ translated from the good epoxy drop substrate images $I_{g}$ using the CycleGAN network with different loss functions as the cycle consistency loss. The number of generated images for each loss function separately and in combination was 1408.

From Table 1, one can see that the $L_{2}$ loss received the lowest score for all the metrics when used alone and performed better when used in combination with LPIPS and the SSIM. The standard cycle consistency loss function $L_{1}$ loss performed worse than both the SSIM and LPIPS. These findings correlate with the visual examination of the images shown in Figure 8. We also found that combining the SSIM and LPIPS with $L_{1}$ separately improved their scores, but combining all three together (i.e., $L_{1}$ + SSIM + LPIPS) gave the best results and visually looked more realistic and similar to the real rejected images.

Furthermore, from the results of Table 1, one can see that $L_{1}$ performed better than $L_{2}$ and the output generated from the $L_{2}$ function had the blurring effect (see Figure 8), as mentioned in [34]. Additionally, combining $L_{1}$ and $L_{2}$ with the other loss functions also improved the performance of the model, as noted in [32]. It can be seen that applying the SSIM for $L_{1}$ and $L_{2}$ increased the performance of the model. LPIPS as the loss function was found to work better independently as well as in combination since it enhanced the image quality and helped to generate more realistic images, as noted in [45].

### 4.2. Impact of Generated Images on Defect Identification

#### 4.2.1. Identification Metrics

The image classifier ResNet18 [42] was used here as a typical classifier to show the impact of the generated images when performing defect identification. As normally done for classification problems, the confusion matrix, precision, recall, and accuracy of the classifier were found with and without using the generated images. Table 2 shows a depiction of the confusion matrix with precision, recall, and accuracy denoted by

(19) $$Precision=\frac{TP}{TP+FP}$$

(20) $$Recall=\frac{TP}{TP+FN}$$

(21) $$Accuracy=\frac{TP+TN}{TP+TN+FP+FN}$$

where *TP* (true positive) indicates when a rejected image is placed in the defective or rejected class, *TN* (true negative) indicates when a good or non-defective image is placed in the non-defective or good class, *FP* (false positive) indicates when a non-defective or good image is placed in the defective or rejected class, and *FN* (false negative) indicates when a defective or rejected image is placed in the non-defective or good class.

#### 4.2.2. Datasets

To ready the dataset for the classification experiments, we randomly selected 1400 real good epoxy drop substrate images and labeled them as non-defective or good. For the defective or rejected class, the 16 available real defective or rejected epoxy drop substrate images were used to generate 2800 synthesized defective or rejected epoxy drop substrate images (1400 images were generated by the standard loss function CycleGAN and 1400 images were generated by our enhanced loss function CycleGAN). Then, these datasets were divided into 60% training, 20% validation, and 20% testing subsets with no overlap among them.

#### 4.2.3. Identification Outcomes

While keeping the same non-defective or good class images the same, for the defective or rejected class, the classifier ResNet18 was trained in three different ways, as follows:

1. By using real rejected epoxy drop substrate images;
2. By using rejected epoxy drop substrate images and generated rejected epoxy drop substrate images based on the standard loss function CycleGAN;
3. By using rejected epoxy drop substrate images and generated rejected epoxy drop substrate images based on our enhanced loss function CycleGAN.

Then, the above trained models were tested via the same testing data subset whose rejected class consisted of a combination of real and generated rejected epoxy drop substrate images. Table 3 shows a comparison of the identification outcomes. As can be seen from this table, the addition of the synthesized images significantly improved the identification outcome. Furthermore, our enhanced loss function CycleGAN provided a higher identification outcome compared to the standard loss function CycleGAN.

## 5. Conclusions

In this paper, the loss function of the generative adversarial network of CycleGAN was enhanced or modified to generate high-quality defective epoxy drop images for die attachment in IC manufacturing. Such images are needed for the purpose of training or testing vision-based deep neural network inspection systems. A CycleGAN network with different cycle consistency loss functions was designed to generate different sets of synthesized images. Based on three evaluation metrics, it has been shown that by incorporating the loss functions of learned perceptual image patch similarity (LPIPS) and the structural similarity index metric (SSIM) into the standard CycleGAN loss function, more realistic or higher-quality synthesized epoxy drop images are generated as compared to using the CycleGAN standard loss function. Furthermore, it has been shown that our enhanced loss function CycleGAN as a data augmentation approach leads to improved identification outcomes when using a typical image classifier. The enhancement approach developed in this paper is general purpose in the sense that it can be applied to other data augmentation scenarios involving other types of images.

## Figures and Tables

**Table 1:** Image quality evaluation metrics for different loss functions as the cycle consistency loss.

$\mathrm{Loss}\;\mathrm{Function}\;\mathrm{as}\;L_{Cycle}$	PSNR	UQI	VFI
	Mean ± STD	Mean ± STD	Mean ± STD
$L_{1}$ (Standard Cycle GAN)	18.77 ± 4.88	0.86 ± 0.16	0.19 ± 0.08
$L_{2}$	16.65 ± 1.66	0.89 ± 0.09	0.08 ± 0.05
SSIM	21.06 ± 2.23	0.93 ± 0.05	0.20 ± 0.06
LPIPS	22.51 ± 4.23	0.94 ± 0.09	0.26 ± 0.02
$L_{1}$ + SSIM	22.94 ± 4.50	0.93 ± 0.07	0.24 ± 0.02
$L_{2}$ + SSIM	23.39 ± 4.46	0.93 ± 0.08	0.25 ± 0.02
$L_{1}$ + LPIPS	23.26 ± 3.48	0.93 ± 0.07	0.32 ± 0.03
$L_{2}$ + LPIPS	22.24 ± 4.98	0.90 ± 0.15	0.27 ± 0.02
$L_{2}$ + SSIM + LPIPS	20.81 ± 6.75	0.90 ± 0.10	0.17 ± 0.02
$L_{1}$ + SSIM + LPIPS	29.86 ± 5.11	0.98 ± 0.01	0.44 ± 0.03

**Table 2:** Confusion Matrix.

		Correct Labels	
		Rejected	Good
Predicted Labels	Rejected	TP	FP
	Good	FN	TN

**Table 3:** Confusion matrix and identification outcomes for ResNet18 as a typical image classifier.

Data Augmentation Method	Confusion Matrix				Identification Metrics		Accuracy
			Correct Labels		Precision	Recall	
			Rejected	Good			
No augmentation (only real data)	Predicted Labels	Rejected	23	02	0.92	0.04	36%
		Good	540	278			
CycleGAN standard with loss function	Predicted Labels	Rejected	381	05	0.99	0.68	78%
		Good	182	275			
CycleGAN with enhanced loss function	Predicted Labels	Rejected	458	13	0.97	0.81	86%
		Good	105	267			

## Data Availability

Data available on request due to privacy restrictions. Contact the authors.

## References

[1] Li Y., Wong C.P. (2006). Recent advances of conductive adhesives as a lead-free alternative in electronic packaging: Materials, processing, reliability, and applications. Mater. Sci. Eng. R Rep..

[2] Capili M.D. (2019). Understanding die attach epoxy open time. Int. Res. J. Adv. Eng. Sci..

[3] Shorten C., Khoshgoftaar T.M. (2019). A survey on image data augmentation for deep learning. J. Big Data.

[4] Goodfellow I., Pouget-Abadie J., Mirza M., Xu B., Warde-Farley D., Ozair S., Courville A., Bengio Y. Generative Adversarial Nets. Proceedings of the 27th International Conference on Neural Information Processing Systems—Volume 2.

[5] Zhu J.-Y., Park T., Isola P., Efros A.A. Unpaired Image-to-Image Translation using Cycle-Consistent Adversarial Networks. Proceedings of the 2017 IEEE International Conference on Computer Vision (ICCV).

[6] Zhang R., Isola P., Efros A.A., Shechtman E., Wang O. The Unreasonable Effectiveness of Deep Features as a Perceptual Metric. Proceedings of the 2018 IEEE/CVF Conference on Computer Vision and Pattern Recognition.

[7] Wang Z., Bovik A.C., Sheikh H.R., Simoncelli E.P. (2004). Image quality assessment: From error visibility to structural similarity. IEEE Trans. Image Process..

[8] Antoniou A., Storkey A., Edwards H., Kůrková V., Manolopoulos Y., Hammer B., Iliadis L., Maglogiannis I. (2018). Augmenting Image Classifiers Using Data Augmentation Generative Adversarial Networks. Proceedings of the 27th International Conference on Artificial Neural Networks.

[9] Chen Y., Yang X.-H., Wei Z., Heidari A.A., Zheng N., Li Z., Chen H., Hu H., Zhou Q., Guan Q. (2022). Generative adversarial networks in medical image augmentation: A review. Comput. Biol. Med..

[10] Sampath V., Maurtua I., Aguilar Martín J.J., Iriondo A., Lluvia I., Aizpurua G. (2023). Intraclass image augmentation for defect detection using generative adversarial neural networks. Sensors.

[11] Wen L., Wang Y., Li X. (2022). A New Cycle-consistent adversarial networks with attention mechanism for surface defect classification with small samples. IEEE Trans. Ind. Inf..

[12] Wang R., Hoppe S., Monari E., Huber M.F. Defect Transfer GAN: Diverse Defect Synthesis for Data Augmentation. Proceedings of the 33rd British Machine Vision Conference (BMVC 2022).

[13] Yang B., Liu Z., Duan G., Tan J. (2022). Mask2Defect: A prior knowledge-based data augmentation method for metal surface defect inspection. IEEE Trans. Ind. Inf..

[14] Niu S., Li B., Wang X., Peng Y. (2022). Region- and Strength-Controllable GAN for defect generation and segmentation in industrial images. IEEE Trans. Ind. Inf..

[15] Hu J., Yan P., Su Y., Wu D., Zhou H. (2021). A method for classification of surface defect on metal workpieces based on twin attention mechanism generative adversarial network. IEEE Sens. J..

[16] Zhang H., Pan D., Liu J., Jiang Z. (2022). A novel MAS-GAN-based data synthesis method for object surface defect detection. Neurocomputing.

[17] Zhang G., Cui K., Hung T.-Y., Lu S. Defect-GAN: High-Fidelity Defect Synthesis for Automated Defect Inspection. Proceedings of the 2021 IEEE Winter Conference on Applications of Computer Vision (WACV).

[18] Jain S., Seth G., Paruthi A., Soni U., Kumar G. (2022). Synthetic data augmentation for surface defect detection and classification using deep learning. J. Intell. Manuf..

[19] Du Z., Gao L., Li X. (2023). A new contrastive GAN with data augmentation for surface defect recognition under limited data. IEEE Trans. Instrum Meas..

[20] He Y., Song K., Dong H., Yan Y. (2019). Semi-supervised defect classification of steel surface based on multi-training and generative adversarial network. Opt. Lasers Eng..

[21] Niu S., Li B., Wang X., Lin H. (2022). Defect image sample generation with GAN for improving defect recognition. IEEE Trans. Autom. Sci. Eng..

[22] Zhai W., Zhu J., Cao Y., Wang Z. A Generative Adversarial Network Based Framework for Unsupervised Visual Surface Inspection. Proceedings of the 2018 IEEE International Conference on Acoustics, Speech and Signal Processing (ICASSP).

[23] Lai Y.T.K., Hu J.S., Tsai Y.H., Chiu W.Y. Industrial Anomaly Detection and One-class Classification using Generative Adversarial Networks. Proceedings of the 2018 IEEE/ASME International Conference on Advanced Intelligent Mechatronics (AIM).

[24] Lu H.-P., Su C.-T. (2021). CNNs combined with a conditional GAN for mura defect classification in TFT-LCDs. IEEE Trans. Semicond. Manuf..

[25] Liu J., Zhang B.G., Li L. Defect detection of fabrics With Generative Adversarial Network Based flaws modeling. Proceedings of the 2020 Chinese Automation Congress (CAC).

[26] Qian W., Xu Y., Li H. (2022). A self-sparse generative adversarial network for autonomous early-stage design of architectural sketches. Comput. Aided Civ. Inf..

[27] Xu Y., Tian Y., Li H. (2023). Unsupervised deep learning method for bridge condition assessment based on intra-and inter-class probabilistic correlations of quasi-static responses. Struct. Health Monit..

[28] Chen K., Cai N., Wu Z., Xia H., Zhou S., Wang H. (2023). Multi-scale GAN with transformer for surface defect inspection of IC metal packages. Expert Syst. Appl..

[29] Li J., Cai N., Mo Z., Zhou G., Wang H. (2021). IC solder joint inspection via generator-adversarial-network based template. Mach. Vis. Appl..

[30] Chen S.-H., Kang C.-H., Perng D.-B. (2020). Detecting and measuring defects in wafer die using GAN and YOLOv3. Appl. Sci..

[31] Jeon Y., Kim H., Lee H., Jo S., Kim J., Ghosh A., Wei L.-Y. (2022). GAN-based Defect Image Generation for Imbalanced Defect Classification of OLED panels. Proceedings of the Eurographics Symposium on Rendering 2022.

[32] Abu-Srhan A., Abushariah M.A.M., Al-Kadi O.S. (2022). The effect of loss function on conditional generative adversarial networks. J. King Saud Univ.-Comput. Inf. Sci..

[33] Pathak D., Krahenbuhl P., Donahue J., Darrell T., Efros A.A. Context Encoders: Feature Learning by Inpainting. Proceedings of the 2016 IEEE Conference on Computer Vision and Pattern Recognition (CVPR).

[34] Isola P., Zhu J., Zhou T., Efros A. Image-to-Image Translation with Conditional Adversarial Networks. Proceedings of the 2017 IEEE Conference on Computer Vision and Pattern Recognition (CVPR).

[35] Abobakr A., Hossny M., Nahavandi S. SSIMLayer: Towards Robust Deep Representation Learning via Nonlinear Structural Similarity. Proceedings of the 2019 IEEE International Conference on Systems, Man and Cybernetics (SMC).

[36] Zhao H., Gallo O., Frosio I., Kautz J. (2017). Loss functions for image restoration with neural networks. IEEE Trans. Comput. Imaging.

[37] Shao G., Huang M., Gao F., Liu T., Li L. (2020). DuCaGAN: Unified dual capsule generative adversarial network for unsupervised image-to-image translation. IEEE Access.

[38] Niu S., Li B., Wang X., He S., Peng Y. (2022). Defect attention template generation CycleGAN for weakly supervised surface defect segmentation. Pattern Recognit..

[39] Krizhevsky A., Sutskever I., Hinton G.E. (2017). ImageNet classification with deep convolutional neural networks. Commun. ACM.

[40] Yamins D.L., DiCarlo J.J. (2016). Using goal-driven deep learning models to understand sensory cortex. Nat Neurosci..

[41] Li C., Wand M., Leibe B., Matas J., Sebe N., Welling M. (2017). Precomputed Real-Time Texture Synthesis with Markovian Generative Adversarial Networks. Proceedings of the Computer Vision—ECCV 2016.

[42] He K., Zhang X., Ren S., Sun S. Deep Residual Learning for Image Recognition. Proceedings of the IEEE Conference on Computer Vision and Pattern Recognition (CVPR).

[43] Wang Z., Bovik A.C. (2002). A universal image quality index. IEEE Signal Process. Lett..

[44] Sheikh H.R., Bovik A.C. (2006). Image information and visual quality. IEEE Trans. Image Process..

[45] Jo Y., Yang S., Kim S.J. Investigating Loss Functions for Extreme Super-Resolution. Proceedings of the 2020 IEEE/CVF Conference on Computer Vision and Pattern Recognition Workshops (CVPRW).

